# The impact of symptoms and illness insight on treatment adherence in first-episode psychosis: a one-year follow-up study using therapeutic drug monitoring

**DOI:** 10.1186/s12888-026-08301-9

**Published:** 2026-06-19

**Authors:** Juhani Leijala, Olli Kampman, Teemu Gunnar, Jaana Suvisaari

**Affiliations:** 1 Department of Clinical Medicine (Psychiatry), Faculty of Medicine, University of Turku, Turku, Finland; 2 Department of Psychiatry, The Wellbeing Services County of South Ostrobothnia, Seinäjoki, Finland; 3 Finnish Institute for Health and Welfare, Mental Health Team, Helsinki, Finland; 4 Department of Psychiatry, The Wellbeing Services County of Ostrobothnia, Vaasa, Finland; 5 Department of Clinical Sciences, Psychiatry, Umeå University, Umeå, Sweden; 6 Department of Psychiatry, The Wellbeing Services County of Pirkanmaa, Tampere, Finland; 7 Forensic Chemistry Unit, Finnish Institute for Health and Welfare, Helsinki, Finland; 8 Department of Psychiatry, South Ostrobothnia Wellbeing Services County, Hanneksenrinne 7, Seinäjoki, 60220 Finland

**Keywords:** Treatment adherence, Therapeutic drug monitoring, Insight, Schizophrenia, Positive symptoms, Antipsychotic medication, Longitudinal study

## Abstract

**Background:**

Despite extensive research on adherence in first-episode psychosis (FEP), reliance on self-report or clinician judgment has limited accuracy and obscured nuanced clinical associations.

**Objective:**

This longitudinal study examined the predictive value of psychopathology, insight, and functioning for objectively measured treatment adherence across a one-year follow-up using therapeutic drug monitoring (TDM).

**Methods:**

Seventy-eight FEP patients were assessed at baseline, 2 months, and 12 months. Clinical measures included the Brief Psychiatric Rating Scale (BPRS), Scale for the Assessment of Negative Symptoms (SANS), Social and Occupational Functioning Assessment Scale (SOFAS), and the Schedule for the Assessment of Insight–Expanded version (SAI-E). Adherence was defined by comparing plasma antipsychotic concentrations to prescribed doses according to international consensus guidelines. Generalized linear mixed models (GLMMs) were used to model time-dependent associations.

**Results:**

While overall adherence declined, time alone was not a significant predictor. Instead, current positive symptom severity (approximately 4–5% lower odds of adherence per BPRS point) was associated with lower odds of adherence, while prior non-adherence predicted subsequent adherence. In contrast, insight, negative symptoms, and functioning showed no consistent association with adherence. A marginal association between improved functioning and better adherence was observed at 12 months.

**Conclusions:**

Adherence in FEP appears to fluctuate in relation to current clinical state rather than follow a stable linear pattern. These findings highlight the value of objective TDM monitoring and early intervention, supporting predictive models that incorporate evolving symptom patterns rather than static baseline measures.

**Trial registration:**

Clinical trial number: not applicable.

**Supplementary Information:**

The online version contains supplementary material available at https://doi.org/10.1186/s12888-026-08301-9.

## Background

Medication adherence during the first year of treatment in first-episode psychosis (FEP) is a dynamic process that may vary substantially over time in relation to changes in psychopathology, insight, and functional capacity [1, 2]. Although poor adherence has been associated with adverse outcomes such as relapse, rehospitalization, and functional decline, identifying clinically meaningful predictors of adherence in early psychosis remains challenging, particularly in settings characterized by intensive treatment and close follow-up [3, 4]. Previous research suggests that individual-level clinical factors may influence adherence trajectories, but their relative importance and temporal stability during the early phases of illness are not well established [5, 6].

Insight has frequently been identified as a key psychological determinant of medication adherence in psychotic disorders, with several studies suggesting that impaired insight increases the risk of treatment discontinuation [7–9]. However, findings have been heterogeneous, and insight–adherence associations have often been weak or nonsignificant in early psychosis, where insight levels fluctuate and intensive clinical follow-up may buffer their behavioral impact [10, 11]. These inconsistencies highlight the need for longitudinal studies using objective adherence measures to clarify the clinical relevance of insight in the early phases of illness.

Despite extensive research on adherence in FEP, most studies have relied on self-reported measures or clinical impressions, which are prone to recall bias and often overestimate adherence rates [1, 12]. Objective assessments such as therapeutic drug monitoring (TDM) provide a more accurate and reliable measure by comparing observed plasma antipsychotic concentrations with levels expected from the prescribed dose [13, 14]. However, few longitudinal studies have applied TDM to examine how clinical variables, including symptoms and insight, predict adherence over time in FEP.

This study addresses this gap by investigating longitudinal changes in psychopathology, insight, and functioning, and their relationship to treatment adherence across a one-year follow-up in FEP. Using TDM as an objective measure of adherence, we hypothesized that treatment adherence would vary over time and that psychopathology, insight, and functional capacity would be associated with adherence during follow-up.

## Methods

### Participants

**Fig. 1:**
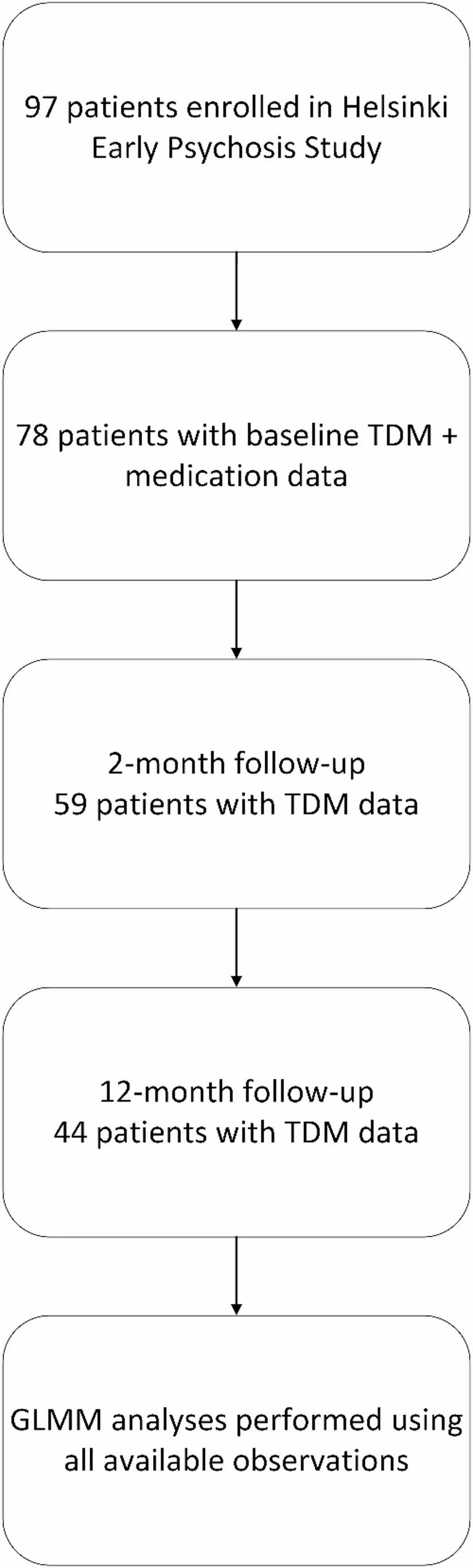
Participant flow and availability of TDM data across follow-up. Flow chart illustrating participant inclusion and availability of therapeutic drug monitoring (TDM) data across the one-year follow-up

The Helsinki Early Psychosis Study (December 2010–July 2016) enrolled 97 adults with FEP, of whom 78 had baseline blood samples and medication data available for TDM-based assessment of treatment adherence. Participants were recruited from both inpatient and outpatient services within the Helsinki and Uusimaa Hospital District, as well as municipal hospital and outpatient clinics in Helsinki. Clinical assessments were conducted at baseline (after treatment initiation and informed consent), and at follow-up visits at 2 and 12 months [15, 16]. Participant flow and availability of TDM data across follow-up are illustrated in Fig. 1.

Diagnostic assessment was conducted using the Structured Clinical Interview for DSM-IV Axis I Disorders, Research Version (SCID-I) [17], and was supplemented by a comprehensive review of medical records covering the entire 12-month follow-up period. Medication use was assessed through prescription review and patient interviews. If participants were unable to recall medication details, data were verified from medical records, including dosage adjustments. In cases where patients reported discontinuing medication, self-reported information was prioritized over medical documentation.

Clinical interviews and symptom assessments were conducted by experienced psychiatric nurses and psychologists with prior clinical and research experience in psychosis assessment. All interviewers received standardized training and ongoing supervision coordinated by J.S., who had completed formal BPRS trainer certification by Joseph Ventura and contributed to the Finnish translation and implementation of the BPRS rating manual. Difficult rating cases were reviewed jointly throughout the study period to maintain consistency across assessors.

Sex was obtained from medical records and reflects biological sex assigned at birth. Information on gender identity or gender diversity was not collected, and therefore analyses are based solely on biological sex.

The study was conducted in accordance with the World Medical Association’s Declaration of Helsinki and was approved by the Ethics Committee of the Helsinki and Uusimaa Hospital District (257/12/03/03/2009), as well as the institutional review boards of the Finnish Institute for Health and Welfare (THL) and the University of Helsinki. Written informed consent was obtained from all participants.

### Measures

Psychopathology was assessed using the Brief Psychiatric Rating Scale, Expanded version (24-items; BPRS) [18]. Negative symptoms were evaluated with the Scale for the Assessment of Negative Symptoms (SANS) [19], functioning with the Social and Occupational Functioning Assessment Scale (SOFAS) [17], and insight with the Schedule for the Assessment of Insight – Expanded Version (SAI-E) [20]. Antipsychotic dosage was calculated using the Defined Daily Dose (DDD) method [21, 22]. Plasma concentrations of antipsychotic medications were measured from blood samples typically drawn the morning after the interview.

Treatment adherence was assessed by comparing measured plasma concentrations with levels expected from the prescribed antipsychotic dose, following the Consensus Guidelines for Therapeutic Drug Monitoring in Neuropsychopharmacology [13]. Based on this comparison, a binary variable (TDM_Adherence) was created to indicate adherence status (adherent vs. non-adherent). TDM_Adherence served as the primary outcome variable in all analyses. An additional variable, Prior_Adherence, indicated adherence at the previous time point and was used to evaluate its predictive value for subsequent adherence.

For more detailed analyses, a five-factor model of the BPRS was used to assess specific symptom dimensions [23, 24]. The model comprised the following subscales: Affect (anxiety, depression, suicidality, guilt); Positive symptoms (grandiosity, suspiciousness, hallucinations, unusual thought content); Negative symptoms (blunted affect, emotional withdrawal, motor retardation); Activation (elated mood, excitement, distractibility, motor hyperactivity); and Disorganization (self-neglect, disorientation, conceptual disorganization, mannerisms and posturing). Additionally, a separate variable, BPRS Positive Worst, which was asked about and rated separately in the interviews, was included to capture the most severe expression of positive symptoms during the acute phase of illness.

### Laboratory analytical methods

Therapeutic drug monitoring analyses were performed at the Forensic Chemistry Unit of the Finnish Institute for Health and Welfare (THL). Plasma antipsychotic concentrations were determined using validated ultra-high performance liquid chromatography-triple quadrupole-tandem mass spectrometry (UHPLC–QQQ–MS/MS) methodology as previously described [14, 25]. Sample preparation was performed using liquid–liquid extraction, and quantification was carried out by multiple reaction monitoring using calibration curves and internal standardization in each analysis batch. The antipsychotics quantified and their limits of quantification (LOQs) are reported in the earlier publication [14]. All samples were stored at − 80 °C until analysis.

### Statistical methods

Statistical analyses were performed in IBM SPSS Statistics for Windows, Version 29.0 [26]. Variable distributions were assessed for normality, and non-parametric methods were applied where assumptions were not met. Group differences were tested with the Mann–Whitney U test for continuous variables and Fisher’s exact test for categorical variables. A dropout analysis compared baseline characteristics between participants who completed follow-up and those lost to follow-up.

Adherence transitions were visualized in RStudio (Version 2022.7.2.576) using an alluvial (Sankey) diagram [27]. At each visit (baseline, 2 months, 12 months) patients were classified as Adherent, Non-adherent, or Missing (no TDM/medication data at that visit; re-entry possible). Band widths are proportional to the number of patients (n), flows are colored by baseline status, and strata are labeled with n. We summarized visit-wise distributions, pairwise transitions among patients with non-missing data at both time points (baseline→2 months; 2→12 months), numbers moving into missing out of the full cohort (*N* = 97), and frequencies of complete three-step trajectories.

For the primary analyses, generalized linear mixed models (GLMMs; logit link) predicted binary treatment adherence (TDM_Adherence: 0 = non-adherent, 1 = adherent) across three time points (T1 = baseline, T2 = 2 months, T3 = 12 months). Patient ID (EPID) was modeled as a random intercept to account for within-subject correlation. An autoregressive AR(1) covariance structure was specified and its parameter (Rho) tested. Time was modeled categorically. Time-varying clinical measures (BPRS, SOFAS, SANS, SAI-E) entered the model as concurrent predictors of adherence at the same visit. Prior_Adherence, defined as adherence at the preceding visit, was included as a lagged predictor of subsequent adherence. Models were estimated using maximum likelihood under a missing-at-random assumption, with all available observations incorporated. Model fit was evaluated using the Akaike Information Criterion (AIC), bias-corrected AIC (AICc), Bayesian Information Criterion (BIC), and − 2 log-likelihood (− 2LL). Explanatory power was summarized using marginal and conditional R². Regression coefficients (β), odds ratios (ORs) with 95% confidence intervals (CIs), and two-sided p-values are reported.

Because cross-sectional contrasts and GLMM results differed, we performed exploratory post hoc analyses. Change scores were defined as baseline − follow-up (and 2 months − 12 months for the later interval); thus, ΔBPRS > 0 indicates symptom reduction and ΔSOFAS < 0 indicates functional improvement. We compared ΔBPRS and ΔSOFAS between adherent and non-adherent groups at 2 and 12 months using two-sided two-sample t-tests (Mann–Whitney U as a sensitivity analysis). Pearson and Spearman correlations quantified associations between change scores and adherence at concurrent and subsequent time points. All post hoc analyses were complete-case and unadjusted, reflecting their exploratory intent.

## Results

The study sample consisted of 78 individuals experiencing FEP (Table 1). The mean age was 26.9 years (SD = 5.9), and 66.7% were male. On average, symptom severity at the baseline assessment was moderate, as reflected by a mean BPRS score of 43.2 (SD = 10.3). Functioning, assessed with the SOFAS, was markedly impaired (mean = 40.2, SD = 8.6). Nearly all participants (97.4%) received antipsychotic medication, with a mean Defined Daily Dose (DDD) of 1.2 (SD = 0.8). At baseline, 60.3% of patients were hospitalized. The most common diagnoses were schizophrenia (35.9%), schizophreniform disorder (26.9%), and unspecified psychotic disorder (12.8%).

**Table 1:** Baseline sociodemographic, clinical, and service use characteristics by follow-up status

Variables	Total (*n* = 78)	Lost to follow-up (*n* = 37)	Completed follow-up (*n* = 41)	*p*-value
	Median [25th–75th percentile] or *n* (%)			
Age	25.4 [22.4–30.3]	25.3 [22.3–34.1]	25.4 [22.6–29.0]	0.492
Sex (male)	52 (66.7%)	31/37 (83.8%)	21/41 (51.2%)	0.004*
Married or cohabitating	15 (19.2%)	8/37 (21.6%)	7/41 (17.1%)	0.775
Employed or student	49 (63.6%)	23/37 (62.2%)	26/40 (65.0%)	0.817
BPRS	42.5 [36.8–48.3]	43.0 [34.0-48.5]	42.0 [37.0-48.5]	0.763
Defined Daily Dose	1.0 [0.6–1.5]	1.00 [0.6–1.9]	1.00 [0.7–1.5]	0.806
SAI-E	15.0 [12.0–18.0]	15.0 [12.0–18.0]	14.5 [9.8–18.0]	0.666
SOFAS	40.0 [35.0–42.0]	40.0 [35.0–40.0]	40.0 [35.0-43.5]	0.64
SANS	1.3 [0.8-2.0]	1.3 [0.6–2.1]	1.3 [0.8-2.0]	0.3
Patient on antipsychotic medication	76 (97.4%)			
Outpatient visits, year 1	25.5 [15.8–31.0]	26.0 [16.5–30.5]	25.0 [15.5–32.5]	0.772
Inpatient episodes ≥ 1, year 1	59/78 (75.6%)	27/37 (73.0%)	32/41 (78.0%)	0.792
Inpatient days, year 1	18.5 [0.75–42.5]	21.0 [0.0–38.0]	16.0 [2.0–50.0]	0.661
Diagnoses (DSM-IV)	78 (100%)			
Substance-induced psychotic disorder	2 (2.6%)			
Schizophrenia	28 (35.9%)			
Schizophreniform disorder	21 (26.9%)			
Schizoaffective disorder	2 (2.6%)			
Bipolar disorder type I	7 (8.9%)			
Psychotic depression	4 (5.1%)			
Delusional disorder	1 (1.3%)			
Brief psychotic disorder	3 (3.8%)			
Psychotic disorder not otherwise specified	10 (12.8%)			
Hospitalized at baseline	47 (60.3%)			
Adherent (Based on TDM)	44 (56.4%)			

*Statistically significant (*p* < 0.05), with moderate effect size (*r* = 0.344)

Values are presented as median [25th–75th percentile] or n (%). Continuous variables were compared using the Mann–Whitney U test, and categorical variables using Fisher’s exact test. BPRS, Brief Psychiatric Rating Scale; SAI-E, Schedule for the Assessment of Insight – Expanded Version; SANS, Scale for the Assessment of Negative Symptoms; SOFAS, Social and Occupational Functioning Assessment Scale; TDM, therapeutic drug monitoring

A comparison between participants lost to follow-up (*n* = 37) and those who completed the follow-up (*n* = 41) revealed no statistically significant or clinically meaningful differences in baseline characteristics, except for sex (Table 1). Males were significantly more likely to be lost to follow-up (83.8% vs. 51.2%, *p* = 0.004).

### Comparison of adherence groups across time points

At baseline, treatment adherence was significantly associated with hospitalization status: 72.7% of adherent patients had been hospitalized, compared to 44.1% of non-adherent patients (*p* = 0.019) (Table 2). No significant differences were found in other background variables, including age, sex, symptom severity (BPRS), functioning (SOFAS), antipsychotic dose (DDD), negative symptoms (SANS), or insight (SAI-E).

**Table 2:** Comparison of adherent and non-adherent patients at different time points

Variable	BL adherent (*n* = 44)	BL non-adherent (*n* = 34)	*p*
	Mean or *n* (%, S.D.)		
Age, mean	27.9 (6.3)	25.7 (5.2)	0.17
Sex (male)	27 (61.4%)	25 (73.5%)	0.335
Hospitalized	32 (72.7%)	15 (44.1%)	**0.019**
BPRS	44.3 (10.4)	41.8 (10.0)	0.326
Defined Daily Dose	1.3 (0.9)	1.1 (0.6)	0.8
SAI-E	14.1 (6.4)	14.2 (4.7)	0.63
SOFAS	39.7 (7.3)	40.8 (10.1)	0.949
SANS	1.4 (0.9)	1.3 (0.8)	0.557
	2 m adherent (*n* = 29)	2 m non-adherent (*n* = 30)	
Sex (male)	17 (58.4)	17 (56.7)	1.0
Hospitalized	3 (10.3)	5 (16.7)	0.706
BPRS	38.0 (11.4)	34.3 (9.2)	0.141
Defined Daily Dose	1.3 (0.7)	1.1 (0.5)	0.146
SAI-E	15.4 (6.5)	17.8 (5.2)	0.105
SOFAS	44.7 (10.1)	48.5 (13.2)	0.406
SANS	1.6 (1.1)	1.1 (1.0)	0.099
	12 m adherent (*n* = 18)	12 m non-adherent (*n* = 26)	
Sex (male)	10 (55.6)	13 (50.0)	0.767
Hospitalized	2 (11.1)	0 (0.0)	0.165
BPRS	39.0 (11.4)	29.8 (5.2)	**0.003**
Defined Daily Dose	1.5 (0.9)	0.9 (0.5)	**0.029**
SAI-E	18.0 (5.5)	18.0 (4.7)	0.583
SOFAS	46.8 (10.6)	61.2 (16.6)	**0.005**
SANS	2.0 (0.9)	1.0 (0.9)	**0.001**

Values are presented as mean (standard deviation) for continuous variables and n (%) for categorical variables. Group comparisons were conducted at baseline (BL), 2 months (2 m), and 12 months (12 m) follow-up based on adherence status determined by therapeutic drug monitoring (TDM). The Mann–Whitney U test was used for continuous variables, and Fisher’s exact test for categorical variables. Statistically significant p-values (*p* < 0.05) are shown in bold. BPRS, Brief Psychiatric Rating Scale; SAI-E, Schedule for the Assessment of Insight – Expanded Version; SANS, Scale for the Assessment of Negative Symptoms; SOFAS, Social and Occupational Functioning Assessment Scale; TDM, Therapeutic Drug Monitoring

At the 2-month follow-up, no statistically significant differences were observed between adherent and non-adherent patients in terms of sex, hospitalization, symptom severity (BPRS), functioning (SOFAS), insight (SAI-E), negative symptoms (SANS), or antipsychotic dose (DDD). However, adherent patients tended to have slightly higher BPRS scores and more pronounced negative symptoms, while non-adherent patients showed somewhat better functioning and higher insight. These differences did not reach statistical significance.

At the 12-month follow-up, adherent patients had significantly higher BPRS scores and received higher antipsychotic doses, while non-adherent patients showed better global functioning as measured by SOFAS. Negative symptoms (SANS) were also more pronounced among adherent patients (all *p* < 0.05).

**Fig. 2:**
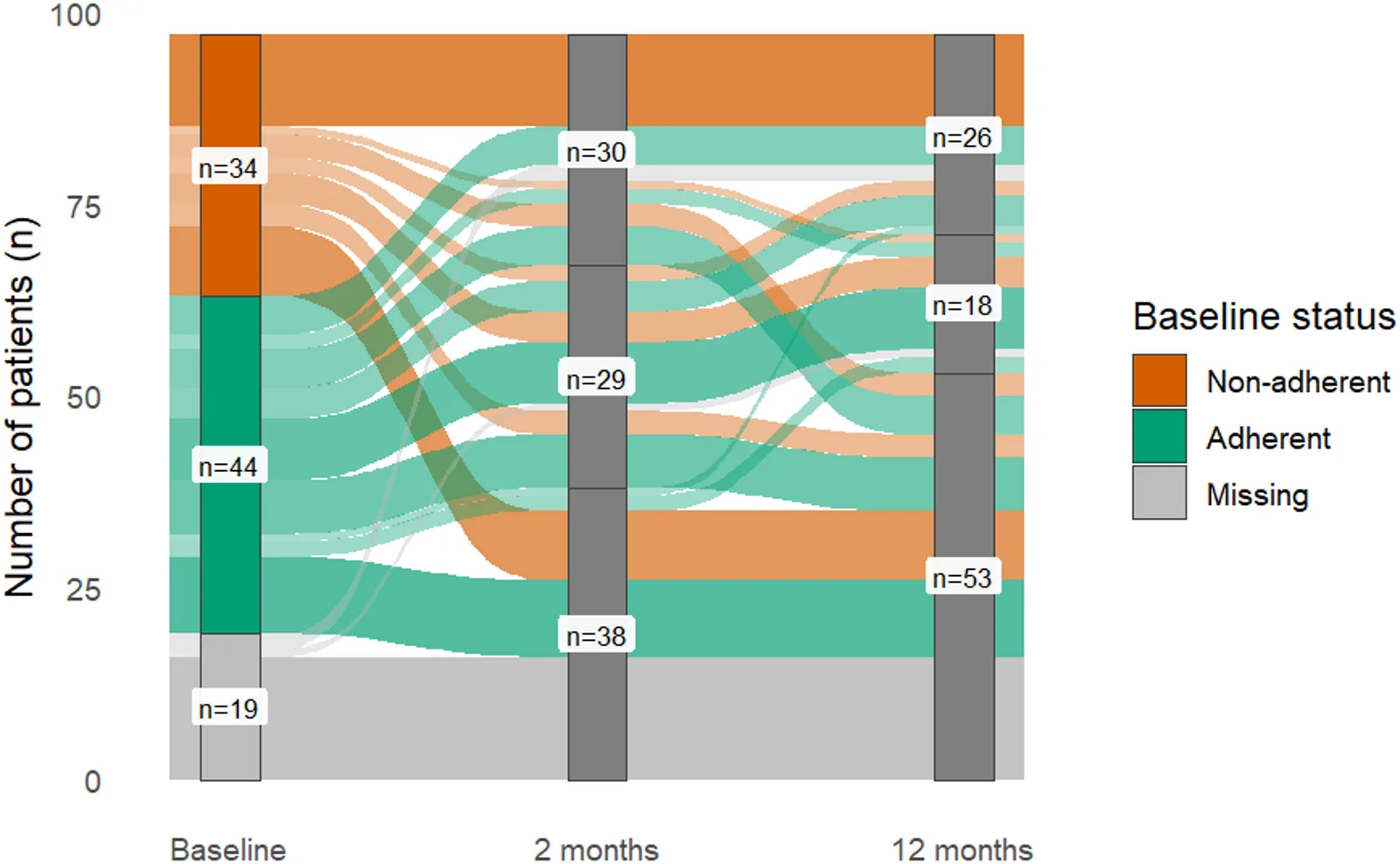
Sankey plot of TDM-defined adherence trajectories (baseline→2 months→12 months). TransitionsThis paragraph is the footnote/legend for Fig. 2 and should be moved under Fig. 2. Please remove it from this location. are shown for the full cohort (N = 97). Baseline TDM was available for 78 patients. Pairwise percentages use only patients with non-missing data at both time points; movements into Missing are out of N = 97. Interpretation: thicker within-state bands indicate persistence; thicker flows to Missing indicate visit-level dropout. Baseline→2 months (n = 56): remained 62.5% (Adherent→Adherent 19; Non-adherent→Non-adherent 16), switched 37.5% (Adherent→Non-adherent 12; Non-adherent→Adherent 9); plus 22/97 (22.7%) moved into Missing. 2→12 months (n = 41): remained 78.0% (Non-adherent→Non-adherent 19; Adherent→Adherent 13), switched 22.0% (Adherent→Non-adherent 6; Non-adherent→Adherent 3); plus 18/97 (18.6%) moved into Missing. Complete 3-step (n = 38): Non-adherent→Non-adherent→Non-adherent 12 (31.6%); Adherent→Adherent→Adherent 8 (21.1%). Missing, no TDM/medication data at that visit (visit-level dropout; re-entry possible); n, number of patients; TDM, Therapeutic Drug Monitoring

Figure 2 shows individual-level adherence transitions for the full cohort (*N* = 97; Missing = no TDM/medication data at that visit). From baseline→2 months, among patients with data at both time points (*n* = 56), 62.5% remained in the same category and 37.5% switched; additionally, 22/97 (22.7%) moved into Missing. From 2→12 months (*n* = 41), 78.0% remained and 22.0% switched; a further 18/97 (18.6%) moved into Missing.

### Comparison of generalized linear mixed models for treatment adherence

Among all models tested (Supplement 1), those including Prior_Adherence provided the best fit. The interaction model (TIME * Prior_Adherence) showed the highest explanatory power and lowest AICc and BIC values, followed closely by the main effects model (TIME + Prior_Adherence). In contrast, models based on baseline clinical variables demonstrated substantially weaker fit.

In models excluding Prior_Adherence, random variance was statistically significant in most cases, indicating that inter-individual differences contributed meaningfully to adherence outcomes. Temporal correlation (AR (1) Rho) was also consistently significant (*p* < 0.001) in these models, confirming a clear time-dependent pattern in adherence. When Prior_Adherence was included, AR (1) Rho was no longer significant (*p* = 0.262), indicating that prior adherence accounted for most of the observed autocorrelation.

### Main effect model results

In the main effect models (Supplement 2), higher overall symptom severity (BPRS) was significantly associated with lower treatment adherence (OR = 0.96, *p* = 0.038), indicating a 4% decrease in the odds of adherence for each one-point increase in BPRS score. Prior adherence was a strong predictor: patients who had been non-adherent at the previous time point had over four times higher odds of being adherent at the next assessment (OR = 4.14, *p* = 0.004). SOFAS and SANS showed trends toward significance, suggesting that better functioning and fewer negative symptoms may be linked to improved adherence. Insight (SAI-E) was not significantly associated with adherence.

In the main effects models, the Positive and Negative subcomponents of the BPRS showed trends toward lower adherence, though neither reached statistical significance. Each point increase in BPRS Negative was associated with a 37% reduction in adherence odds (OR = 0.63), and in BPRS Positive with a 24% reduction (OR = 0.76). Other BPRS domains - Affect, Activation, Disorganization, and Positive Worst - were not associated with adherence. Time alone had no significant effect.

### Temporal development of treatment adherence and influencing factors

**Fig. 3:**
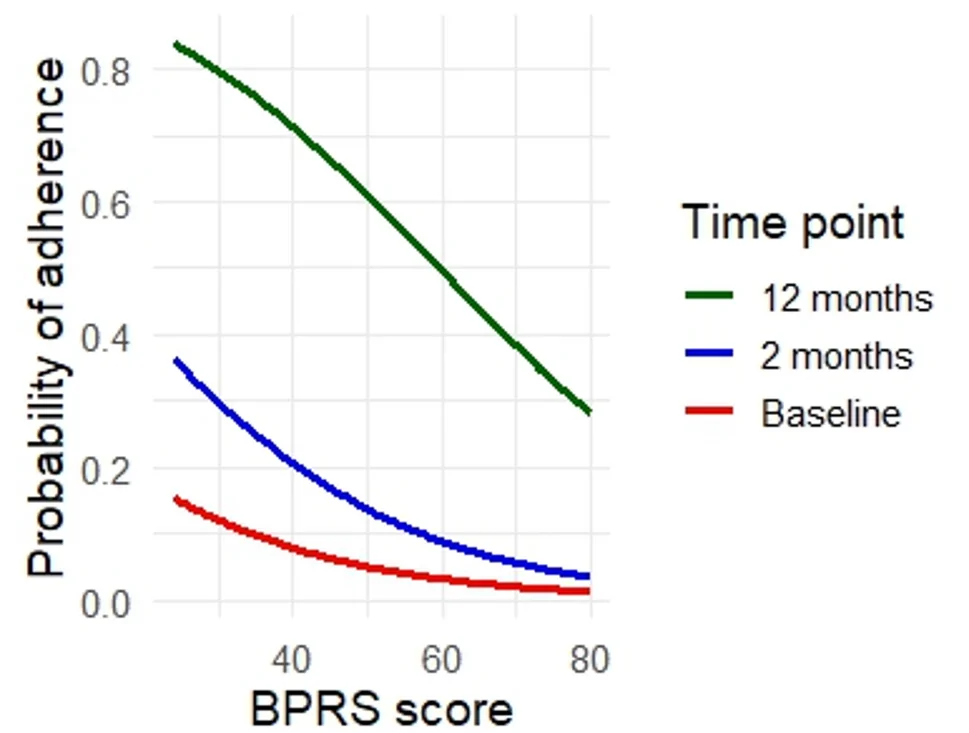
Predicted probability of treatment adherence by symptom severity (BPRS) at different time points. Modeled probabilities were estimated using a generalized linear mixed model with a TIME*BPRS interaction. Higher BPRS scores were consistently associated with lower predicted adherence probabilities at baseline, 2 months, and 12 months

In the interaction models (Table 3), higher total BPRS scores were significantly associated with lower treatment adherence at baseline and 2 months. Each one-point increase in BPRS corresponded to a 4–5% decrease in the odds of adherence (OR = 0.96, *p* = 0.019; OR = 0.95, *p* = 0.039). At 12 months, the association was not statistically significant but showed a similar trend (OR = 0.95, *p* = 0.061). Figure 3 illustrates the predicted probability of adherence across BPRS scores: a 10-point increase in BPRS corresponded to approximately 34% lower odds of adherence at baseline (OR₁₀ = 0.66) and 40% lower odds at 2 months (OR₁₀ = 0.60), reducing a predicted adherence probability of 70% to about 61% and 58%, respectively. Higher symptom severity was thus consistently linked to reduced adherence probability at all time points. At 12 months, patients who had been non-adherent at 2 months had 11.5 times the odds of adherence compared with those adherent at 2 months (OR = 11.5, *p* = 0.003). At 2 months, prior non-adherence at baseline was associated with higher odds of adherence, but this did not reach statistical significance (OR = 3.29, *p* = 0.075).

**Table 3:** Interaction effects between time and clinical predictors of treatment adherence (GLMM Results)

Model	Predictor	B (SE)	*p*	OR (95% CI)
TIME*SAI-E	SAI * [T1]	-0.001 (0.040)	0.977	1.00 (0.92–1.08)
	SAI * [T2]	0.025 (0.036)	0.488	1.03 (0.96–1.10)
	SAI * [T3]	0.038 (0.036)	0.291	1.04 (0.97–1.12)
TIME*BPRS	BPRS * [T1]	-0.046 (0.019)	**0.019**	0.96 (0.92–0.99)
	BPRS * [T2]	-0.049 (0.024)	**0.039**	0.95 (0.91–1.00)
	BPRS * [T3]	-0.048 (0.025)	0.061	0.95 (0.91–1.00)
TIME*SOFAS	SOFAS * [T1]	0.025 (0.020)	0.221	1.03 (0.99–1.07)
	SOFAS * [T2]	0.026 (0.018)	0.147	1.03 (0.99–1.06)
	SOFAS * [T3]	0.031 (0.016)	**0.048**	1.03 (1.00–1.07)
TIME*SANS	SANS * [T1]	-0.390 (0.243)	0.11	0.68 (0.42–1.09)
	SANS * [T2]	-0.315 (0.250)	0.21	0.73 (0.45–1.20)
	SANS * [T3]	-0.337 (0.260)	0.196	0.71 (0.43–1.19)
TIME*Prior_Adherence	[T2]*[PriorAdherence = 0]	1.191 (0.662)	0.075	3.29 (0.89–12.24)
	[T2]*[PriorAdherence = 1]	0.255 (0.626)	0.684	1.29 (0.37–4.47)
	[T3]*[PriorAdherence = 0]	2.439 (0.808)	**0.003**	11.5 (2.30–57.1)
	[T3]*[PriorAdherence = 1]	0b	.	.
TIME*BPRS_Affect	Affect*[T1]	-0.263 (0.178)	0.14	0.77 (0.54–1.09)
	Affect*[T2]	-0.155 (0.212)	0.465	0.86 (0.56–1.30)
	Affect*[T3]	-0.171 (0.242)	0.479	0.84 (0.52–1.36)
TIME*BPRS_Positive	Positive * [T1]	-0.314 (0.144)	**0.031**	0.73 (0.55–0.97)
	Positive * [T2]	-0.460 (0.229)	**0.046**	0.63 (0.40–0.99)
	Positive * [T3]	-0.569 (0.310)	0.068	0.57 (0.31–1.04)
TIME*BPRS_Negative	Negative * [T1]	-0.535 (0.302)	0.078	0.59 (0.32–1.06)
	Negative * [T2]	-0.435 (0.315)	0.169	0.65 (0.35–1.21)
	Negative * [T3]	-0.346 (0.327)	0.29	0.71 (0.37–1.35)
TIME*BPRS_Activation	Activation * [T1]	0.286 (0.760)	0.707	1.33 (0.30–5.96)
	Activation * [T2]	0.533 (0.854)	0.533	1.71 (0.32–9.19)
	Activation * [T3]	0.817 (0.886)	0.358	2.26 (0.39–13.02)
TIME*BPRS_Disorganization	Disorganization * [T1]	-0.665 (0.513)	0.197	0.51 (0.19–1.42)
	Disorganization * [T2]	-0.406 (0.523)	0.439	0.67 (0.24–1.87)
	Disorganization * [T3]	-0.269 (0.525)	0.609	0.76 (0.27–2.15)
TIME*BPRS_Positive_Worst	Positive_Worst * [T1]	-0.150 (0.130)	0.249	0.86 (0.67–1.11)
	Positive_Worst * [T2]	-0.147 (0.162)	0.367	0.86 (0.63–1.19)
	Positive_Worst * [T3]	-0.138 (0.214)	0.52	0.87 (0.57–1.33)

TIME indicates the measurement points: T1 (baseline, 0 months), T2 (2 months), and T3 (12 months, reference). Interaction terms (TIME*Variable) indicate that the effect of the predictor on adherence varies over time. Significant interaction terms suggest that the relationship between the predictor and adherence changes at different time points. B (SE) represents the beta coefficient and standard error, showing the effect size and its uncertainty. p denotes the statistical significance, with values below 0.05 considered significant. OR (95% CI) shows the odds ratio and confidence interval, where OR > 1 indicates increased odds, OR < 1 indicates decreased odds, and OR = 1 indicates no change. PRIOR_Adherence reflects previous adherence status: 0 (non-adherent) and 1 (adherent, reference). BPRS, Brief Psychiatric Rating Scale (subscales: Affect, Positive, Negative, Activation, Disorganization, Positive_Worst); B (SE), Beta coefficient (Standard Error); OR, Odds Ratio; p, p-value; PRIOR_Adherence, Prior adherence status; SAI, Schedule for the Assessment of Insight – Expanded Version; SANS, Scale for the Assessment of Negative Symptoms; SE, Standard Error; SOFAS, Social and Occupational Functioning Assessment Scale

Among the BPRS subcomponents, only positive symptoms showed a significant time-dependent association with adherence. At baseline, each point increase in positive symptoms reduced the odds of adherence by 27% (OR = 0.73, *p* = 0.031), and at 2 months by 37% (OR = 0.63, *p* = 0.046). A marginal association was also found between improved functioning (SOFAS) and better adherence at 12 months (OR = 1.03, *p* = 0.048). No significant time-dependent effects were observed for other symptom dimensions (Affect, Activation, Disorganization, Positive Worst, or Negative symptoms), nor for insight (SAI-E) or negative symptoms (SANS).

Group differences in ΔBPRS and ΔSOFAS were non-significant in both intervals (all two-sided *p* ≥ 0.15; Mann-Whitney concordant), with small effects. One association emerged: greater early symptom improvement (ΔBPRS baseline→2 months) correlated with lower adherence at 12 months (Spearman ρ = -0.33, *p* = 0.03; Pearson *r* = -0.32, *p* = 0.04); all other correlations were non-significant (*p* ≥ 0.12).

## Discussion

This study investigated treatment adherence over a one-year follow-up in first-episode psychosis and assessed the influence of psychopathology, insight, and functioning. Objective therapeutic drug monitoring (TDM) provided more accurate and reliable adherence estimates compared with previous self-report–based studies.

This analysis complements our previous work on the same cohort, which focused on comparing different adherence assessment methods [14]. In contrast to that earlier methodological evaluation, the present study examines adherence as a dynamic, longitudinal outcome and identifies clinical predictors of objectively measured adherence.

Although adherence declined over the one-year follow-up, consistent with earlier reports in FEP [11, 28], time itself was not a significant predictor. Instead, current symptom severity and prior adherence determined changes in adherence, indicating that adherence patterns are dynamic rather than uniformly declining. The use of GLMMs captured both within-subject changes and between-subject differences, revealing associations that would be obscured in simpler cross-sectional analyses.

Positive symptoms at assessment were associated with poorer adherence, whereas the retrospective worst-rating of positive symptoms (BPRS Positive Worst) was not, underscoring the importance of current symptomatology. This lack of association for the Positive Worst rating further reinforces that concurrent symptoms, rather than peak historical severity, predicted adherence. When modeled longitudinally, insight, functioning, and negative symptoms showed only weak or inconsistent associations.

The AR (1) correlation showed that adherence was linked across time points, but not in a stable or linear way. Patients who were non-adherent at 2 months were frequently adherent at 12 months, indicating that adherence behavior can fluctuate substantially rather than persistently decline. Including prior adherence in the model eliminated random variance and autocorrelation, suggesting that recent adherence status explains most of the temporal structure. This finding contrasts with earlier reports of stable persistence [29, 30] and may reflect intensified clinical responses after early non-adherence, including closer follow-up, treatment modifications, or other interventions commonly used in early psychosis care [31]. Regression to the mean and the inherent variability of early psychosis may also contribute to this pattern. In addition, the association between symptom severity and adherence is likely bidirectional: worsening symptoms may reduce adherence, while non-adherence itself may contribute to symptom exacerbation and relapse. This interpretation is consistent with qualitative studies describing the dynamic and evolving nature of antipsychotic treatment experiences in early psychosis [32].

The alluvial visualization (Fig. 2) complements the model-based results by showing that status persistence was more common than switching, particularly from 2 to 12 months, while early switching from adherent to non-adherent also occurred. The increasing missing stratum illustrates visit-level dropout and underscores the need to interpret cross-sectional contrasts with attrition in mind. Consistent with this pattern, including prior adherence in the GLMMs largely removed autocorrelation, indicating that recent adherence captures much of the temporal structure. The Sankey pattern is compatible with fluctuating clinical trajectories in which early improvement may be followed by medication discontinuation and later relapse; cross-sectional snapshots can therefore capture different phases of this cycle.

Psychotic symptom severity was the strongest clinical predictor of adherence, with higher BPRS scores linked to poorer adherence. This effect persisted over time and trended toward significance at 12 months. Only positive symptoms, such as delusions and hallucinations, predicted adherence, aligning with evidence that paranoia and mistrust can drive refusal or dropout [33] and that sustained improvement in positive symptoms supports adherence [8, 34]. Early lapses did not necessarily persist, suggesting opportunities for corrective interventions or natural recovery. To avoid confusion, it is important to note upfront that the seemingly contradictory 12-month cross-sectional result (higher BPRS among adherent patients) does not conflict with the longitudinal findings. The apparent contrast with the 12-month cross-sectional comparison likely reflects the influence of prior adherence and cumulative time effects. Longitudinal GLMM modeling thus confirms that higher symptom severity predicts lower adherence over time.

Negative symptoms and functional capacity showed no consistent associations with adherence. A marginal effect suggested that better functioning at 12 months was linked to improved adherence, echoing findings that impaired functioning contributes to long-term disengagement [35, 36]. Their influence may emerge over longer periods or through indirect pathways. Insight was similarly unrelated to adherence, which contrasts with much of the existing literature [8, 9] and suggests that, in early psychosis, intensive follow-up and external support may temporarily buffer the behavioral impact of poor insight. This may partly reflect the Finnish public psychiatric care setting, where patients with FEP are typically treated within integrated inpatient and outpatient services with relatively intensive early follow-up. This divergence highlights how clinical context and follow-up structure shape the behavioral consequences of insight. This interpretation aligns with evidence that insight fluctuates considerably during the early treatment phase and may not yet translate into stable medication-taking behavior.

Key strengths of this study include the objective TDM-based adherence assessment, which minimizes bias common to self-report or clinician ratings, and the use of longitudinal GLMM modeling that captured both individual variation and time-dependent effects. Longitudinal FEP studies that combine repeated TDM-based adherence assessments with detailed symptoms and insight measurements are still relatively rare, which underscores the added value of these data for refining early-intervention strategies. Incorporating prior adherence and validated clinical instruments further enhanced explanatory power and robustness. We also explicitly examined dropout effects to address potential attrition bias. Post hoc analyses reinforced the primary conclusion that adherence reflects the current clinical state rather than earlier symptom changes, while the single inverse association between early symptom relief and later adherence may indicate reduced perceived medication need.

Limitations include the modest sample size, which may have reduced statistical power for interaction analyses, and missing data with loss to follow-up, a common issue in psychosis research where clinical instability and psychosocial factors hinder sustained participation [37]. Although GLMMs accommodate incomplete data through maximum likelihood estimation, this approach assumes that data are missing at random. In early psychosis, however, disengagement, symptom exacerbation, and non-adherence often co-occur, raising the possibility that missingness may follow a missing-not-at-random pattern. This may particularly affect estimates at the 12-month follow-up, where patients with worsening symptoms or disengagement from services might have been underrepresented in the available data. Selection bias is possible because patients who completed the 12-month follow-up may represent a subgroup more willing or able to maintain treatment engagement, potentially inflating the observed link between prior non-adherence and later adherence. Similar attrition and engagement effects are well documented in early psychosis cohorts, where disengagement rates of 15–20% are typical [38]. However, service disengagement was not recorded as a separate structured variable in the present dataset, and therefore its relationship with TDM-based adherence could not be formally examined. Unmeasured variables, including cognitive functioning, therapeutic alliance, subjective treatment experiences, side-effect burden, shared decision-making processes, and treatment modifications during the follow-up period, might also have influenced adherence. Such treatment modifications might have included long-acting injectable antipsychotics, compulsory treatment, or intensified adherence-focused interventions. In addition, insight levels in this treated FEP cohort were generally in the moderate-to-good range, which may have restricted variability in SAI-E scores and attenuated potential associations with adherence. Sex was recorded only as biological sex assigned at birth, and gender identity was not assessed, which limits the ability to evaluate potential sex- or gender-related differences in treatment adherence. These limitations should be considered when interpreting the findings, but the longitudinal design, objective TDM-based adherence assessment, and careful follow-up analyses help mitigate their impact.

## Conclusions

Adherence in FEP is dynamic and context-sensitive, with current symptom severity and prior adherence patterns influencing subsequent adherence behavior. These findings highlight the need for proactive monitoring of current psychotic symptoms, particularly positive symptoms, to support adherence in first-episode psychosis. The strong predictive value of prior adherence, combined with improvement after early lapses, points to opportunities for corrective action and the importance of vigilant follow-up. Over time, negative symptoms and reduced functioning may further compromise adherence, indicating a need for sustained rehabilitation efforts. These results emphasize that adherence support is most effective when concentrated in the early treatment months, when symptom fluctuations and behavioral instability are greatest. Overall, adherence in FEP appears context-dependent and is best supported through continuous symptom management and individualized, symptom-informed strategies for long-term care.

## Supplementary Information

Below is the link to the electronic supplementary material.

Supplementary Material 1

## Data Availability

The datasets generated and/or analysed during the current study are not publicly available due to restrictions related to participant privacy and ethical approvals.

## Abbreviations

−2LL: Negative Two Log-Likelihood
2m/12m: 2 Months/12 Months
AIC: Akaike Information Criterion
AICc: Corrected Akaike Information Criterion
APA: American Psychiatric Association
AR(1): First-Order Autoregressive Correlation Structure
B (β): Regression Coefficient
BIC: Bayesian Information Criterion
BL: Baseline
BPRS: Brief Psychiatric Rating Scale
CI: Confidence Interval
Cond. R²: Conditional R-Squared
DDD: Defined Daily Dose
Delta (Δ): Change Score
DSM-IV: Diagnostic and Statistical Manual of Mental Disorders, Fourth Edition
EPID: Patient Identifier (Random Intercept)
FEP: First-Episode Psychosis
GLMM: Generalized Linear Mixed Model
Marg. R²: Marginal R-Squared
OR: Odds Ratio
p: p-Value
Rho (ρ): AR(1) Autocorrelation Parameter
SAI-E: Schedule for the Assessment of Insight – Expanded Version
SANS: Scale for the Assessment of Negative Symptoms
SCID-I: Structured Clinical Interview for DSM-IV Axis I Disorders
SD: Standard Deviation
SE: Standard Error
SOFAS: Social and Occupational Functioning Assessment Scale
SPSS: Statistical Package for the Social Sciences
T1/T2/T3: Measurement Points: Baseline/2 Months/12 Months
TDM: Therapeutic Drug Monitoring
TDM_Adherence: Binary Adherence Variable Based on TDM
THL: Finnish Institute for Health and Welfare
TIME: Measurement Time-Point Variable
WHO: World Health Organization
