# Supplementary material for: The impact of symptoms and illness insight on treatment adherence in first-episode psychosis: a one-year follow-up study using therapeutic drug monitoring
Source: BMC Psychiatry. 2026 Jun 19;26:690. doi: 10.1186/s12888-026-08301-9 (PMC13540956; doi:10.1186/s12888-026-08301-9)
Supplement: Supplementary file 1 [file 12888_2026_8301_MOESM1_ESM.docx]

Supplement 1. Comparison of Generalized Linear Mixed Models (GLMM) for Treatment Adherence: Main Effect and Interaction Models

| **Main Effect Models** | AICc | BIC | -2LL | Marg. R² | Cond. R² | Random Variance | p (Random Variance) | AR1 Rho | p (AR1 Rho) |
| --- | --- | --- | --- | --- | --- | --- | --- | --- | --- |
| TIME | 809.18 | 815.47 | 805.11 | 0.007 | 0.473 | 1.704 | **0.029** | 0.706 | **<0.001** |
| TIME+SAI-E | 745.18 | 751.28 | 741.10 | 0.011 | 0.449 | 1.528 | **0.046** | 0.725 | **<0.001** |
| TIME+BPRS | 796.78 | 803.01 | 792.71 | 0.033 | 0.45 | 1.476 | **0.032** | 0.638 | **<0.001** |
| TIME+SOFAS | 795.63 | 801.87 | 791.56 | 0.026 | 0.434 | 1.403 | **0.037** | 0.657 | **<0.001** |
| TIME+SANS | 789.60 | 795.83 | 785.53 | 0.026 | 0.425 | 1.35 | **0.039** | 0.631 | **0.002** |
| TIME+Prior_Adherence | 429.71 | 434.67 | 425.58 | 0.103 | 0.278 | 0.483 | 0.484 | 0.408 | 0.631 |
| TIME+BPRS_Affect | 791.03 | 797.27 | 786.96 | 0.013 | 0.458 | 1.585 | **0.032** | 0.692 | **<0.001** |
| TIME+BPRS_Positive | 791.24 | 797.48 | 787.17 | 0.021 | 0.444 | 1.476 | **0.031** | 0.635 | **<0.001** |
| TIME+BPRS_Negative | 789.15 | 795.38 | 785.08 | 0.022 | 0.435 | 1.414 | **0.038** | 0.665 | **<0.001** |
| TIME+BPRS_Activation | 787.29 | 793.52 | 783.22 | 0.008 | 0.458 | 1.601 | **0.032** | 0.693 | **<0.001** |
| TIME+BPRS_Disorganization | 788.82 | 795.06 | 784.75 | 0.012 | 0.457 | 1.587 | **0.03** | 0.673 | **<0.001** |
| TIME+BPRS_Positive_Worst | 790.38 | 796.61 | 786.31 | 0.009 | 0.449 | 1.547 | **0.032** | 0.678 | **<0.001** |
|  |  |  |  |  |  |  |  |  |  |
| **Interaction Models** |  |  |  |  |  |  |  |  |  |
| TIME*SAI-E | 757.61 | 763.71 | 753.53 | 0.014 | 0.456 | 1.565 | **0.044** | 0.724 | **<0.001** |
| TIME*BPRS | 811.13 | 817.37 | 807.06 | 0.034 | 0.449 | 1.465 | **0.033** | 0.638 | **<0.001** |
| TIME*SOFAS | 811.51 | 817.75 | 807.44 | 0.027 | 0.438 | 1.421 | **0.036** | 0.656 | **<0.001** |
| TIME*SANS | 790.14 | 796.38 | 786.07 | 0.017 | 0.421 | 1.353 | **0.04** | 0.651 | **<0.001** |
| TIME*Prior_Adherence | 433.04 | 437.97 | 428.91 | 0.16 | 0.248 | 0.231 | 0.701 | 0.592 | 0.262 |
| TIME*BPRS_Affect | 794.22 | 800.46 | 790.15 | 0.011 | 0.459 | 1.597 | **0.032** | 0.695 | **<0.001** |
| TIME*BPRS_Positive | 795.26 | 801.50 | 791.19 | 0.032 | 0.446 | 1.453 | **0.031** | 0.621 | **0.001** |
| TIME*BPRS_Negative | 790.24 | 796.48 | 786.17 | 0.017 | 0.431 | 1.409 | **0.04** | 0.679 | **<0.001** |
| TIME*BPRS_Activation | 787.50 | 793.73 | 783.43 | 0.007 | 0.458 | 1.608 | **0.031** | 0.691 | **<0.001** |
| TIME*BPRS_Disorganization | 789.53 | 795.77 | 785.46 | 0.011 | 0.457 | 1.594 | **0.029** | 0.67 | **<0.001** |
| TIME*BPRS_Positive_Worst | 795.17 | 801.41 | 791.10 | 0.007 | 0.445 | 1.527 | **0.032** | 0.677 | **<0.001** |

All models are generalized linear mixed models (GLMMs) with a binomial distribution and logit link function. The dependent variable is treatment adherence (TDM), and TIME refers to the measurement time point (baseline, 2 months, 12 months). Predictor variables include total and subscale scores of SAI-E (insight), BPRS (symptom severity), SOFAS (functioning), and SANS (negative symptoms). Models with interaction terms assess time-dependent effects. Statistically significant p-values (p < 0.05) are shown in bold. AICc, corrected Akaike Information Criterion; AR1 Rho, autoregressive correlation coefficient (AR(1) structure for repeated measures); BIC, Bayesian Information Criterion; BPRS, Brief Psychiatric Rating Scale (subscales: Affect, Positive, Negative, Activation, Disorganization, Positive_Worst); Cond. R², conditional R-squared (variance explained by both fixed and random effects); -2LL, -2 log likelihood; Marg. R², marginal R-squared (variance explained by fixed effects); OR, Odds Ratio; p, p-value for random effects and AR1 Rho; PRIOR_Adherence, Prior adherence status; Random Variance, variance of the random intercept (EPID); SAI-E, Schedule for the Assessment of Insight – Expanded Version; SANS, Scale for the Assessment of Negative Symptoms; SE, Standard Error; SOFAS, Social and Occupational Functioning Assessment Scale, TDM, Therapeutic Drug Monitoring.

Supplement 2. Main Effects of Time and Clinical Predictors on Treatment Adherence (GLMM Results)

| **Model** | **Predictor** | **B (SE)** | **p** | **OR (95% CI)** |
| --- | --- | --- | --- | --- |
|  |  |  |  |  |
| TIME | T1 | -0.577 (0.434) | 0.185 | 0.56 [0.24 – 1.32] |
|  | T2 | -0.352 (0.454) | 0.439 | 0.70 [0.29 – 1.72] |
| TIME+SAI-E | T1 | -0.630 (0.477) | 0.188 | 0.53 [0.21 – 1.37] |
|  | T2 | -0.381 (0.490) | 0.438 | 0.68 [0.26 – 1.80] |
|  | SAI-E | 0.017 (0.035) | 0.639 | 1.02 [0.95 – 1.09] |
| TIME+BPRS | T1 | -0.148 (0.481) | 0.759 | 0.86 [0.33 – 2.23] |
|  | T2 | -0.262 (0.467) | 0.576 | 0.77 [0.31 – 1.94] |
|  | BPRS | -0.044 (0.021) | **0.038** | 0.96 [0.92 – 1.00] |
| TIME+SOFAS | T1 | -0.138 (0.494) | 0.781 | 0.87 [0.33 – 2.31] |
|  | T2 | -0.117 (0.482) | 0.808 | 0.89 [0.34 – 2.30] |
|  | SOFAS | 0.030 (0.017) | 0.076 | 1.03 [1.00 – 1.07] |
| TIME+SANS | T1 | -0.603 (0.437) | 0.17 | 0.55 [0.23 – 1.30] |
|  | T2 | -0.394 (0.464) | 0.397 | 0.68 [0.27 – 1.69] |
|  | SANS | -0.367 (0.206) | 0.077 | 0.69 [0.46 – 1.04] |
| TIME+Prior_Adherence | T2 | -0.409 (0.462) | 0.379 | 0.67 (0.27–1.66) |
|  | Prior_Adherence=0 | 1.420 (0.482) | **0.004** | 4.14 (1.59–10.76) |
| TIME+BPRS_Affect | T1 | -0.449 (0.445) | 0.314 | 0.64 (0.27–1.54) |
|  | T2 | -0.305 (0.461) | 0.509 | 0.74 (0.30–1.83) |
|  | Affect | -0.197 (0.178) | 0.27 | 0.82 (0.58–1.17) |
| TIME+BPRS_Positive | T1 | -0.156 (0.502) | 0.756 | 0.86 (0.32–2.31) |
|  | T2 | -0.261 (0.466) | 0.576 | 0.77 (0.31–1.93) |
|  | Positive | -0.274 (0.168) | 0.103 | 0.76 (0.55–1.06) |
| TIME+BPRS_Negative | T1 | -0.563 (0.435) | 0.198 | 0.57 (0.24–1.35) |
|  | T2 | -0.349 (0.461) | 0.45 | 0.71 (0.28–1.75) |
|  | Negative | -0.461 (0.278) | 0.099 | 0.63 (0.36–1.09) |
| TIME+BPRS_Activation | T1 | -0.597 (0.439) | 0.176 | 0.55 (0.23–1.31) |
|  | T2 | -0.360 (0.458) | 0.433 | 0.70 (0.28–1.72) |
|  | Activation | 0.403 (0.773) | 0.603 | 1.50 (0.33–6.89) |
| TIME+BPRS_Disorganization | T1 | -0.583 (0.435) | 0.182 | 0.56 (0.24–1.32) |
|  | T2 | -0.379 (0.460) | 0.411 | 0.69 (0.28–1.70) |
|  | Disorganization | -0.499 (0.477) | 0.296 | 0.61 (0.24–1.56) |
| TIME+BPRS_Positive_Worst | T1 | -0.394 (0.508) | 0.439 | 0.67 (0.25–1.84) |
|  | T2 | -0.272 (0.475) | 0.567 | 0.76 (0.30–1.95) |
|  | Positive_Worst | -0.093 (0.150) | 0.537 | 0.91 (0.68–1.23) |

TIME indicates the measurement points: T1 (baseline, 0 months), T2 (2 months), and T3 (12 months, reference). B (SE) represents the beta coefficient and standard error, showing the effect size and its uncertainty. p denotes the statistical significance, with values below 0.05 considered significant. OR (95% CI) shows the odds ratio and confidence interval, where OR > 1 indicates increased odds, OR < 1 indicates decreased odds, and OR = 1 indicates no change. PRIOR_Adherence reflects previous adherence status: 0 (non-adherent) and 1 (adherent, reference). BPRS, Brief Psychiatric Rating Scale (subscales: Affect, Positive, Negative, Activation, Disorganization, Positive_Worst); B (SE), Beta coefficient (Standard Error); OR, Odds Ratio; p, p-value; PRIOR_Adherence, Prior adherence status; SAI-E, Schedule for the Assessment of Insight – Expanded Version; SANS, Scale for the Assessment of Negative Symptoms; SE, Standard Error; SOFAS, Social and Occupational Functioning Assessment Scale, TDM, Therapeutic Drug Monitoring.
